# Repair of gastro-tracheobronchial fistula after esophagectomy for esophageal cancer using intercostal muscle and latissimus dorsi muscle flaps: a case report

**DOI:** 10.1186/s40792-020-00936-4

**Published:** 2020-07-14

**Authors:** Kazushi Miyata, Masahide Fukaya, Masato Nagino

**Affiliations:** Division of Surgical Oncology, Department of Surgery, Nagoya University Graduate School of Medicine, 65 Tsurumai-cho, Showa-ku, Nagoya, 466-8550 Japan

**Keywords:** Gastro-tracheobronchial fistula, Esophagectomy, Muscle flap

## Abstract

**Background:**

Gastro-tracheobronchial fistula after esophagectomy is a rare but life-threatening complication associated with high mortality. Several authors reported postoperative management of tracheobronchial fistula. However, treatment is demanding and challenging, and the strategy is still controversial.

**Case presentation:**

A 64-year-old man underwent thoracoscopic esophagectomy with two-field lymph node dissection and gastric conduit reconstruction by an intrathoracic anastomosis for esophageal cancer at a local hospital in June 2013. After surgery, a gastro-tracheal fistula and a gastro-bronchial fistula of the left main bronchus were diagnosed, and the patient was referred to our hospital for the management of the gastro-tracheobronchial fistula. CT and bronchoscopy and esophagogastroduodenoscopy performed at our hospital revealed that the gastro-bronchial fistula of the left main bronchus was cured by packing with the omentum from the gastric conduit and the gastro-tracheal fistula located 3 cm above the carina remained open. We concluded that the fistula would not resolve without further surgical procedure. However, such an operation was expected to be difficult and to need much time due to severe adhesion among the gastric conduit and/or trachea, bronchus, lung, and chest wall. Therefore, a two-stage operation was planned for safety and outcome certainty. The first operation was performed to close the fistula in October 2013. The gastric conduit was separated from the trachea and resected; then, the fistula was sutured and covered by intercostal muscle and latissimus dorsi muscle flaps. A month after the first operation, reconstruction with pedunculated jejunum was performed via the percutaneous route. The patient’s postoperative course was uneventful.

**Conclusion:**

If the omentum is not observed between the gastric conduit and the tracheobronchus when a gastro-tracheobronchial fistula occurs after esophagectomy, surgeons should perform surgical treatment because conservative treatment is unlikely to cure. During surgery, the use of two types of muscle flaps, such as the intercostal muscle and the latissimus dorsi muscle flaps, is helpful for the closure of gastro-tracheobronchial fistulas.

## Background

Gastro-tracheobronchial fistula after esophagectomy is a rare but life-threatening complication associated with high mortality [1–3]. Postoperative management of tracheobronchial fistulas has been described. However, treatment is demanding and challenging, and the strategy is still controversial [4, 5].

Here, we present a case of a gastro-tracheal fistula after esophagectomy for esophageal cancer, which was successfully treated by using two types of muscle flaps, such as the intercostal muscle and latissimus dorsi muscle flaps.

## Case presentation

**Fig. 1:**
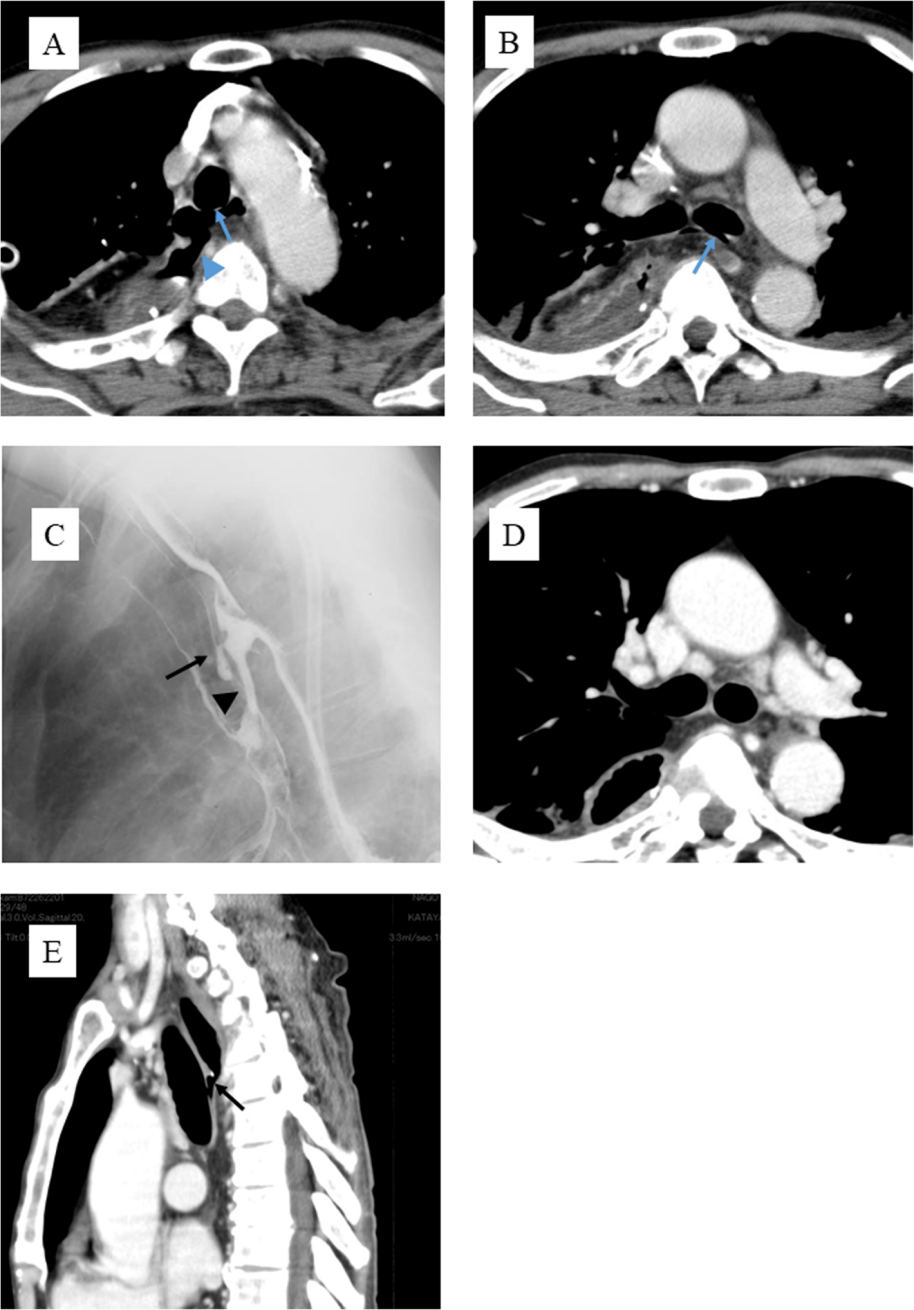
On postoperative day 7, computed tomography (CT) revealed anastomotic leakage (blue arrowhead) and a tracheal fistula (blue arrow) (**a**). On postoperative day 7, a left main bronchial fistula was observed (blue arrow) (**b**). On postoperative day 49, an upper gastrointestinal series showed a fistula within the trachea (black arrow) and a fistula within the left main bronchus (black arrowhead) (**c**). CT (postoperative day 117) revealed that the left main bronchial fistula was closed by covering it with omentum (**d**), and the GTF was open (black arrow) (**e**)

A 64-year-old man underwent esophagectomy for esophageal cancer, with two-field lymph node dissection at a local hospital in June 2013. The alimentary tract was reconstructed using a gastric conduit with an intrathoracic anastomosis. A severe air leak was observed on postoperative day 1. On postoperative day 3, right empyema caused by anastomosis leakage developed. On postoperative day 7, bronchoscopy and computed tomography (CT) revealed tracheal and left main bronchial injury (Fig. 1a, b).

Subsequently, conservative therapy including drainage was performed.

Two months after surgery, a gastro-tracheal fistula and a gastro-bronchial fistula of the left main bronchus were found because an upper gastrointestinal series showed a connection between a gastric conduit and tracheobronchial fistulas (Fig. 1c). After the examination, conservative therapy was continued between 2 months. As a result of the conservative therapy, the gastro-bronchial fistula of the left main bronchus was cured; however, the gastro-tracheal fistula was not cured. Finally, 3 months after surgery, the patient was referred to our hospital for the management of the fistula in September 2013.

**Fig. 2:**
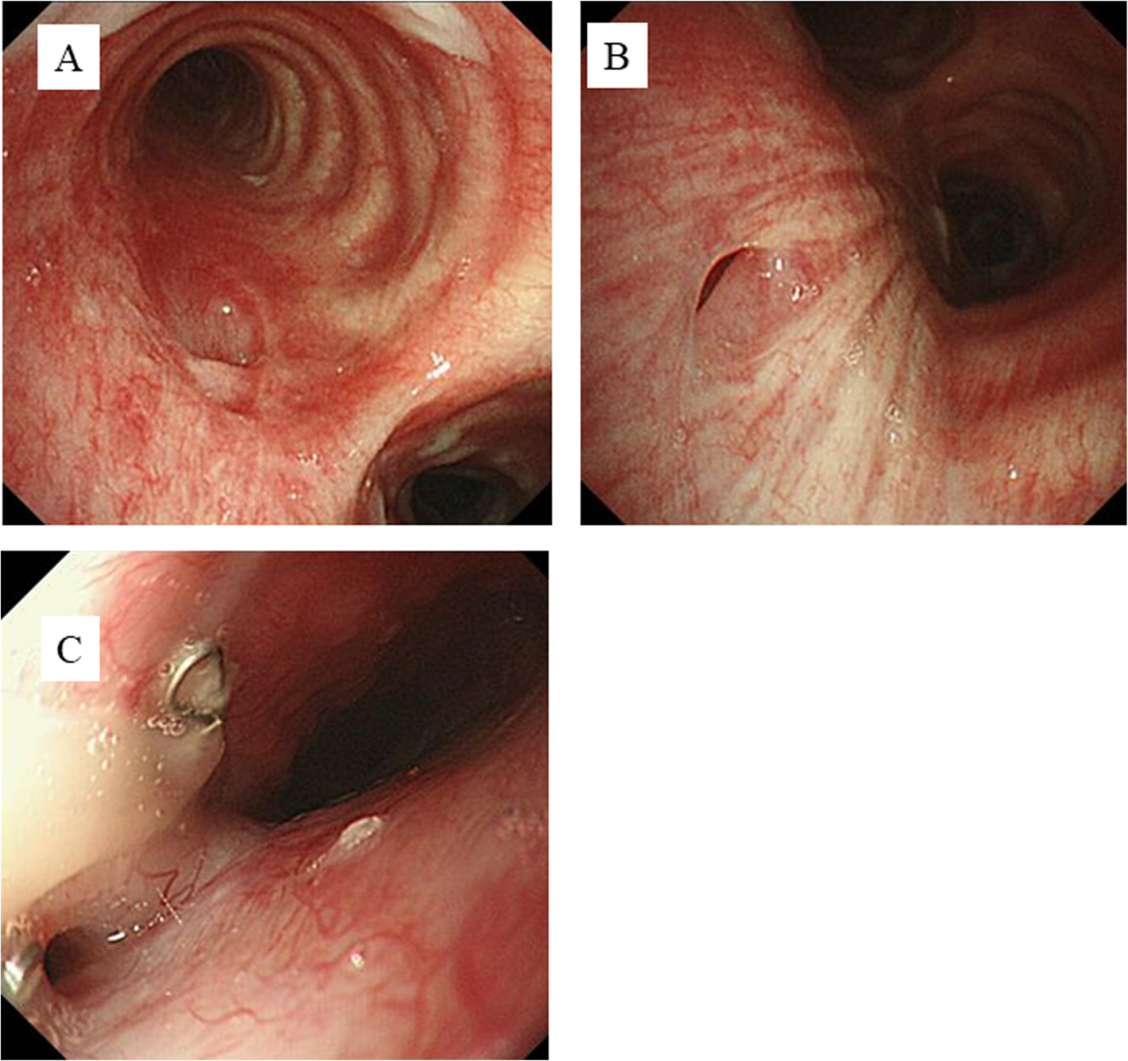
Bronchoscopy revealed that the GBF was covered by mucosa and was cured (**a**). Bronchoscopy revealed that the GTF was located in the trachea, 3 cm above the carina (**b**). Esophagogastroduodenoscopy showed the GTF located in the left wall at the anastomosis (**c**)

CT performed at our hospital revealed that the gastro-bronchial fistula of the left main bronchus was cured by packing with the omentum from the gastric conduit (Fig. 1d), and the gastro-tracheal fistula remained open (Fig. 1e). Bronchoscopy also revealed that the gastro-bronchial fistula of the left main bronchus was closed and covered by mucosa (Fig. 2a) and that the gastro-tracheal fistula was located in the trachea, 3 cm above the carina (Fig. 2b). When swallowing, bubbles of saliva appeared through the fistula. Esophagogastroduodenoscopy showed a fistula located in the left wall at the anastomosis (Fig. 2c). We concluded that the fistula would not resolve without further surgical procedure. However, such an operation was expected to be difficult and to need much time due to severe adhesion among the gastric conduit and/or trachea, bronchus, lung, and chest wall. Therefore, a two-stage operation was planned for safety and outcome certainty.

**Fig. 3:**
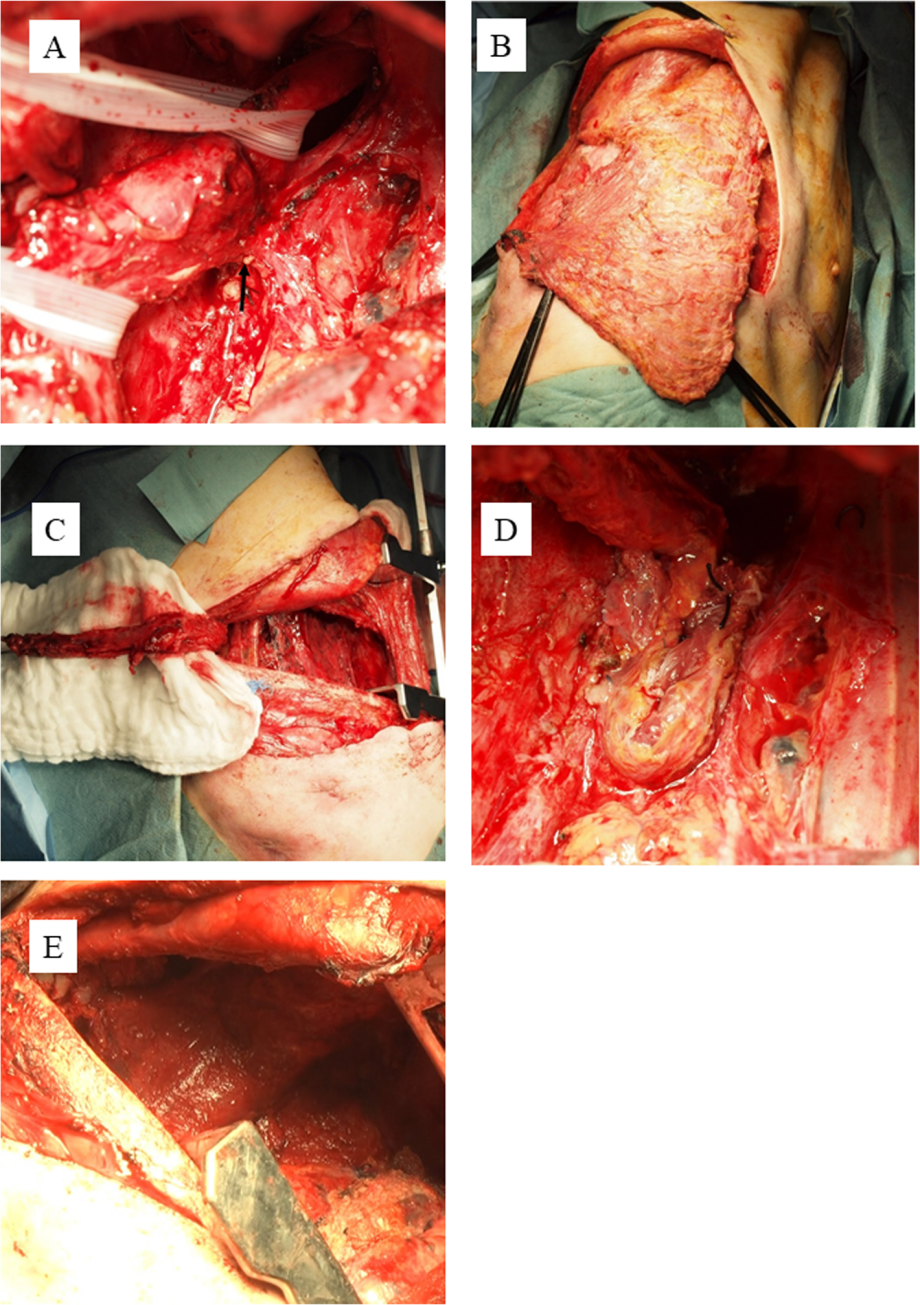
We separated the anastomosis from the trachea. An upper Penrose drain was placed in the esophagus, and a lower Penrose drain was placed in the gastric conduit. The GTF was visualized between the trachea and the gastric conduit (black arrow) (**a**). The latissimus dorsi muscle flap was constructed (**b**). The intercostal muscle flap was constructed (**c**). After incision of the GTF, direct sutures were used to close the fistula, which was then covered using the intercostal muscle flap (**d**). The latissimus dorsi muscle flap covered the area above the intercostal muscle flap (**e**)

**Fig. 4:**
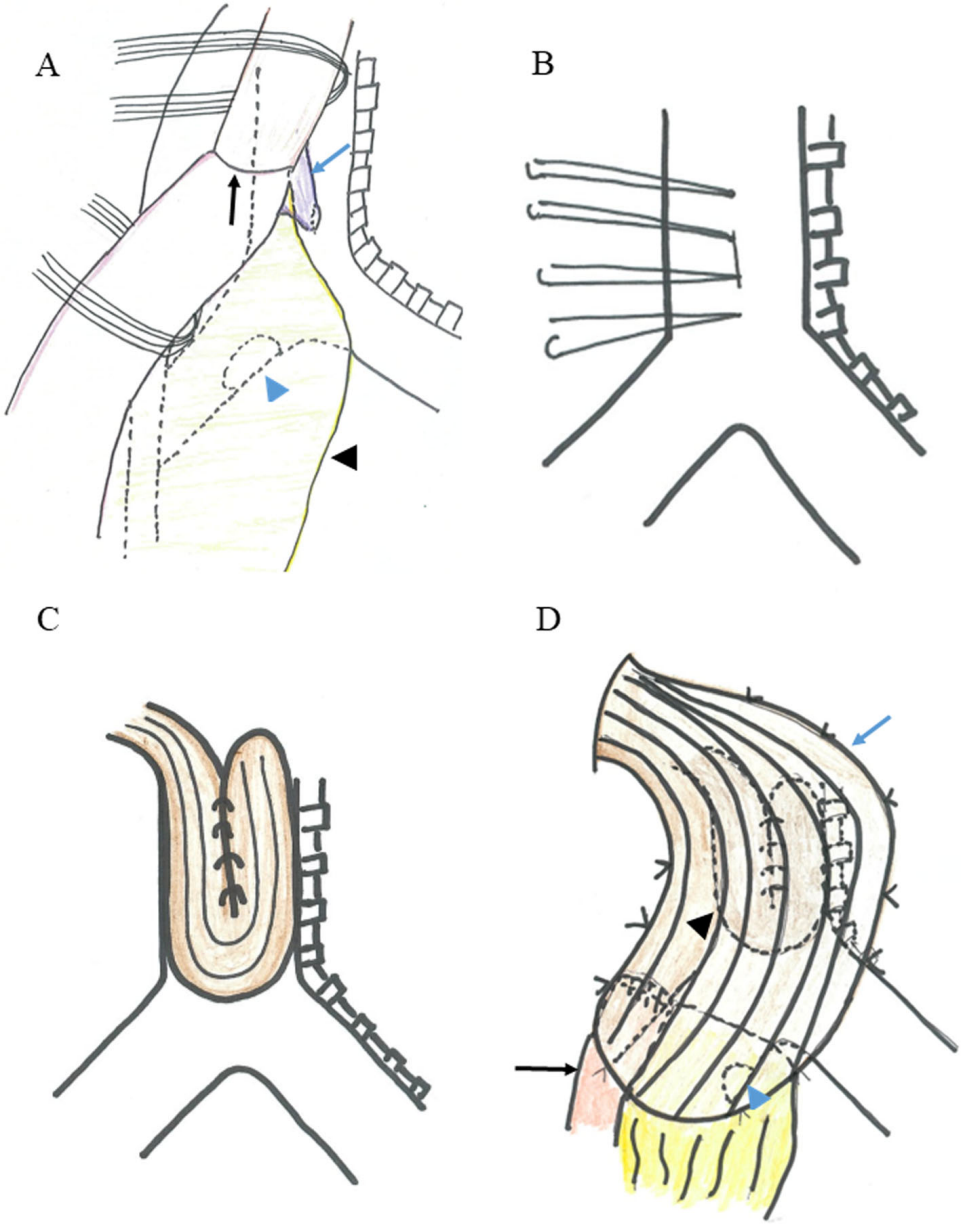
Schema of operative findings. We found anastomosis and separated from the trachea (black arrow). GTF was visualized between the trachea and the gastric conduit (blue arrow). The left main bronchial fistula was covered with omentum (blue arrowhead). Black arrowhead shows the omentum. This schema is created from Fig. 3a (**a**). We performed direct suture for GTF on the right side at the trachea (**b**). The intercostal muscle was inserted and covered above the sutured portion (**c**). The area including the intercostal muscle flap (black arrowhead) and left main bronchial fistula covered with omentum (blue arrowhead) was widely covered and fixed with this latissimus dorsi muscle flap (blue arrow). Black arrow shows the remnant gastric conduit (**d**)

The first operation to close the fistula was performed in October 2013. Though we initially planned to separate the gastric conduit from the trachea and insert muscle flaps into the space, we decided that the gastric conduit should be removed from the right thoracic cavity while preserving the omentum because the wall of the gastric conduit immediately below the esophagogastrostomy had become very thin due to major leakage. However, adhesion was tighter than expected in the right thoracic cavity, and we could not remove the whole gastric conduit while preserving the omentum. The omentum should be preserved for use in covering the gastro-bronchial fistula. Therefore, first, we carefully separated the anastomosis from the trachea and resected the esophagus above the anastomosis and the gastric conduit below the anastomosis. As a result, most of the gastric conduit and the omentum remained, and the fistula was found between the anastomosis and the trachea (Figs. 3a and 4a). Next, direct sutures were placed in the fistula. The fistula was closed with two sutures. In addition to these sutures, another suture was performed at the upper and the lower position of the fistula (Fig. 4b). All ligatures were performed without needle detachment (Fig. 4b). The intercostal muscle flap was constructed and folded side by side to cover the bronchial fistula. The left side of the intercostal muscle flap was sutured from inside to outside followed by the right side from outside to inside (Figs. 3c, d and 4c). Subsequently, the latissimus dorsi muscle flap was constructed and inserted through the second intercostal space. The area including the intercostal muscle flap and left main bronchial fistula covered with omentum was widely covered and fixed with this latissimus dorsi muscle flap (Figs. 3b, e and 4d). After repairing in the thorax, an esophagostomy was performed in the neck.

One month after the first operation, reconstruction with a pedunculated jejunum was performed via the percutaneous route.

The patient’s postoperative course was uneventful and without complications. He was able to start oral intake on postoperative day 18 and was discharged on day 25. He continues to undergo surveillance, and no signs of recurrence have been observed for 6 years after the operation.

## Discussion

A tracheobronchial fistula is classified intraoperatively and postoperatively. In this case, the reason for gastro-tracheobronchial fistula occurrence was unknown because the esophagectomy was performed at the previous hospital where the patient was initially treated. However, one of the possible reasons may be intraoperative tracheobronchial injury because severe air leakage was noted on postoperative day 1. Hulshers et al. [6] reported a tracheobronchial injury rate of 0.8% (1 of 114 patients) for transthoracic esophagectomy, and Maruyama et al. [7] concluded that extensive dissection of more than 60 lymph nodes and three-field lymph node dissections increased the risk of tracheobronchial injury.

In this case, unfortunately, the tracheal fistula and the bronchial fistula were related to gastric conduit leakage because both the fistulas and the leak were near each other. However, the gastro-bronchial fistula of the left main bronchus was cured with conservative therapy during the 2 months after the esophagectomy at the previous hospital. The gastro-tracheal fistula remained open upon subsequent examinations following referral to our hospital. These differences were caused by the height of fistulas because the gastro-bronchial fistula of the left main bronchus was covered with omentum (Fig. 1b), and the gastro-tracheal fistula was uncovered (Fig. 1a). Whether a gastro-tracheobronchial fistula is cured by conservative therapy may depend on the presence of the omentum between the gastric conduit and the tracheobronchus. When performing gastric tube reconstruction by intrathoracic anastomosis, it is necessary to wrap the anastomosis with omentum tightly and insert the omentum between the gastric conduit and the tracheobronchus to prevent gastro-tracheobronchial fistula formation.

Considering the conservative treatment period, Morita et al. [5] concluded that if the tracheobronchial fistula fails to heal within a 4–6-week period, conservative management should be abandoned. In fact, the gastro-tracheal fistula had not been cured during the 16-week period in our case. The optimal treatment for a tracheobronchial fistula is controversial; however, we agree with their opinion and recommend changing fistula therapy if the fistula is not cured after several weeks of conservative treatment. In this case, severe air leakage was recognized on postoperative day 1, and right empyema was caused by anastomosis leakage as soon as postoperative day 3. Furthermore, a low probability of complete recovery existed with conservative therapy because the omentum was not present between the gastric conduit and the trachea on CT on postoperative day 7. Therefore, the surgeons at the previous hospital should have decided to perform surgical intervention earlier.

In this case, we successfully treated gastro-tracheobronchial fistula after esophagectomy using two different muscle flaps. Gastro-tracheobronchial fistula is a rare but life-threatening complication, and the treatment is controversial. A muscle flap has been reported to be useful for covering tracheobronchial fistulas. Though the use of one type of muscle flaps is the general method, we used two types of muscle flaps, namely, intercostal muscle and latissimus dorsi muscle. Intercostal muscle flap interposition sometimes has been reported for bronchial stump reinforcement during a lobectomy [8]; however, this technique has rarely been reported for gastro-tracheobronchial fistula treatment [9]. An intercostal muscle flap is useful for tight closure because it is a narrow muscle, as shown in Fig. 3d. However, it is feared that the intercostal muscle does not have enough thickness to cover the fistula because it is the narrow muscle. A latissimus dorsi muscle is useful for covering wide areas because it is a wide muscle, as shown in Fig. 3e. However, using a latissimus dorsi muscle flap is not suited to cover fistula tightly because it is a wide muscle. Therefore, we used two different types of muscle flaps to supply each shortage.

## Conclusion

If the omentum is not observed between the gastric conduit and the tracheobronchus when a gastro-tracheobronchial fistula occurs after esophagectomy, surgeons should perform surgical treatment because conservative treatment is unlikely to cure. During surgery, the use of two types of muscle flaps, such as the intercostal muscle and the latissimus dorsi muscle flaps, is helpful for the closure of gastro-tracheobronchial fistulas.

## Data Availability

We would not like to share data other than those described in the paper because they include personal information.

## Abbreviation

CT: Computed tomography
